# Publisher Correction: Spin-controlled atom–ion chemistry

**DOI:** 10.1038/s41467-018-04165-0

**Published:** 2018-04-23

**Authors:** Tomas Sikorsky, Ziv Meir, Ruti Ben-shlomi, Nitzan Akerman, Roee Ozeri

**Affiliations:** Department of Physics of Complex Systems, Weizmann Institute of Science, 7610001 Rehovot, Israel

Correction to: *Nature Communications* 10.1038/s41467-018-03373-y, published online 02 March 2018

The original version of this Article contained an error in the third sentence of the first paragraph of the section “Spin polarizing the Sr^+^ ion with ultracold atoms” in Results, which incorrectly read as “The Langevin collision rate is 1”. The correct version adds “kHz” after “1”.

The fifth sentence of this same paragraph originally read as “Although ^87^Rb has a *I* = 3/2 nuclear spin and a hyperfine-split ground-state manifold, ^88^Sr has no nuclear spin and a Zeeman split two-fold ground state”, which is incorrect. The correct version states “^88^Sr^+^” instead of “^88^Sr”.

The first sentence of the fourth paragraph of this same section originally read as “As the collisional energies are on the mK energy scale, spin exchange between Sr^+^ and Rb prepared in the *F* = 1 state is allowed only as long as it does not require Rb to change its hyperfine state and climb the 330 m hyperfine energy gap”, which is incorrect. The correct version states “330 mK” instead of “330 m”.

In the Discussion section, the text was originally incorrectly repeated.

This has been corrected in both the PDF and HTML versions of the Article.

