# Supplementary material for: Spin-controlled atom–ion chemistry
Source: Nat Commun. 2018 Mar 2;9:920. doi: 10.1038/s41467-018-03373-y (PMC5834540; doi:10.1038/s41467-018-03373-y)
Supplement: Supplementary file 1 — Supplementary Information [file 41467_2018_3373_MOESM1_ESM.pdf]

Spin controlled atom-ion chemistry

Sikorsky et al.

Supplementary Information

Supplementary Note 1: **Clebsch-Gordan decomposition**

To obtain the projection to a singlet manifold we expand the atoms hyperfine state to the electronic spin basis.

$$\begin{aligned}\left|\left\langle \langle 1,-1|_{\rm Rb}\otimes\langle\downarrow|_{\rm Sr^+}\middle|\text{singlet}\right\rangle\right|^2 &= \frac{3}{4}\left|\left\langle \langle\tfrac{3}{2},-\tfrac{3}{2}|_{\rm nucl}\otimes\langle\uparrow|_{\rm elec}\otimes\langle\downarrow|_{\rm Sr^+}\middle|\text{singlet}\right\rangle\right|^2 = 0.375\\ \left|\left\langle \langle 1,0|_{\rm Rb}\otimes\langle\downarrow|_{\rm Sr^+}\middle|\text{singlet}\right\rangle\right|^2 &= \frac{1}{2}\left|\left\langle \langle\tfrac{3}{2},-\tfrac{1}{2}|_{\rm nucl}\otimes\langle\uparrow|_{\rm elec}\otimes\langle\downarrow|_{\rm Sr^+}\middle|\text{singlet}\right\rangle\right|^2 = 0.25\end{aligned} \tag{1}$$

When atoms are initialized in a $|1,-1\rangle_{\rm Rb}$ state and ion is collisionally pumped to $P(\downarrow)\sim 0.9$, the projection to a singlet manifold is $0.9\times 0.375+0.1\times 0.25 = 0.3625$. A ratio between the charge-exchange rates of $|1,-1\rangle_{\rm Rb}$ and $|1,0\rangle_{\rm Rb}$ is $\frac{0.3625}{0.25}=1.45$.

To quantitatively describe the spin-exchange we expand the atoms hyperfine state in the electronic spin basis and analyze the collisions in the two-electron basis.

$$\begin{aligned}|1,-1\rangle_{\rm Rb}\otimes|\downarrow\rangle_{\rm Sr^+} &= \underbrace{\frac{\sqrt{3}}{2}\left|\tfrac{3}{2},-\tfrac{3}{2}\right\rangle_{\rm nucl}\otimes\overbrace{|\uparrow\rangle_{\rm elec}\otimes|\downarrow\rangle_{\rm Sr^+}}^{\text{anti}-\text{parallel spins}}}_{\text{spin exchange energetically suppresed}} - \frac{1}{2}\left|\tfrac{3}{2},-\tfrac{1}{2}\right\rangle_{\rm nucl}\otimes\overbrace{|\downarrow\rangle_{\rm elec}\otimes|\downarrow\rangle_{\rm Sr^+}}^{\text{parallel spins}}\\ &\xRightarrow{\text{spin exch.}} \frac{\sqrt{3}}{2}\underbrace{\left|\tfrac{3}{2},-\tfrac{3}{2}\right\rangle_{\rm nucl}\otimes|\downarrow\rangle_{\rm elec}}_{|{\rm F}=2,{\rm m_F}=-2\rangle_{\rm Rb}}\otimes|\uparrow\rangle_{\rm Sr^+} - \frac{1}{2}\left|\tfrac{3}{2},-\tfrac{1}{2}\right\rangle_{\rm nucl}\otimes|\downarrow\rangle_{\rm elec}\otimes|\downarrow\rangle_{\rm Sr^+}\end{aligned} \tag{2}$$

$$\begin{aligned}|1,-1\rangle_{\rm Rb}\otimes|\uparrow\rangle_{\rm Sr^+} &= \frac{\sqrt{3}}{2}\left|\tfrac{3}{2},-\tfrac{3}{2}\right\rangle_{\rm nucl}\otimes\overbrace{|\uparrow\rangle_{\rm elec}\otimes|\uparrow\rangle_{\rm Sr^+}}^{\text{parallel spins}} - \underbrace{\frac{1}{2}\left|\tfrac{3}{2},-\tfrac{1}{2}\right\rangle_{\rm nucl}\otimes\overbrace{|\downarrow\rangle_{\rm elec}\otimes|\uparrow\rangle_{\rm Sr^+}}^{\text{anti}-\text{parallel spins}}}_{\text{spin exchange allowed}}\\ &\xRightarrow{\text{spin exch.}} \frac{\sqrt{3}}{2}\left|\tfrac{3}{2},-\tfrac{3}{2}\right\rangle_{\rm nucl}\otimes|\uparrow\rangle_{\rm elec}\otimes|\uparrow\rangle_{\rm Sr^+} - \frac{1}{2}\underbrace{\left|\tfrac{3}{2},-\tfrac{1}{2}\right\rangle_{\rm nucl}\otimes|\uparrow\rangle_{\rm elec}}_{\frac{1}{\sqrt{2}}|1,0\rangle_{\rm Rb}+\frac{1}{\sqrt{2}}|{\rm F}=2,{\rm m_F}=0\rangle_{\rm Rb}}\otimes|\downarrow\rangle_{\rm Sr^+}\end{aligned} \tag{3}$$

Supplementary Note 2: **Charge-exchange temperature dependence** Using a Doppler cooling thermometry[1] we extracted the ion energy distributions after colliding with atoms prepared in $|2,-2\rangle_{\mathrm{Rb}}$ and $|2,0\rangle_{\mathrm{Rb}}$ manifold. Since these energy distributions are not analytic we define the ions temperature as $k_{\mathrm{B}}T_{\mathrm{ion}} = E_{\mathrm{mode}}/2$, where E_{mode} is the most probable energy[2].

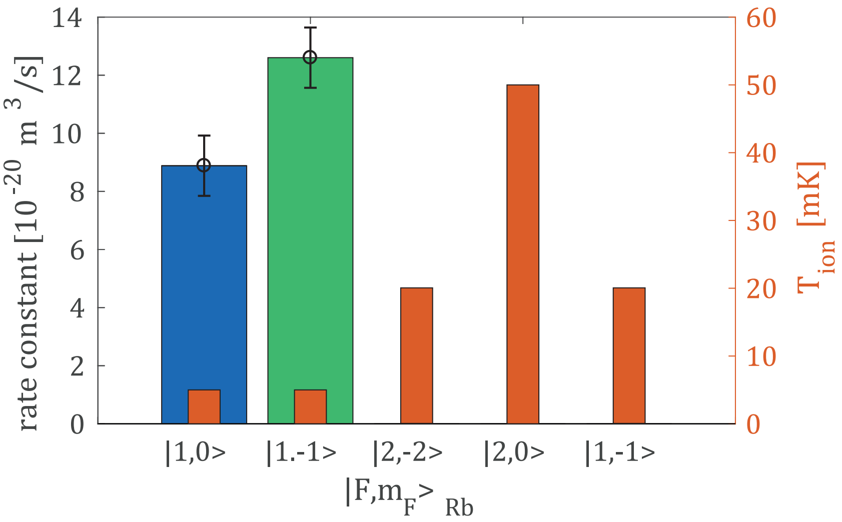

Supplementary Figure 1: **Charge-exchange initial state dependence.** Charge-exchange rate together with temperature of an ion for different initial hyperfine states of Rb atoms. Excess micromotion (EMM) is compensated to a level of ~0.1mK for all but last column. F=2 and $|1,-1\rangle$* column null results are not visible on the chart. The upper one sigma confidence interval for these rate constants is $10^{-20}\,\mathrm{m}^3\,\mathrm{s}^{-1}$. Error bars represent one standard deviation.

Supplementary Note 3: **Charge-exchange YAG power dependence** Even though our experiment is performed in the absence of any resonant laser light, there is $1064\,\mathrm{nm}$ YAG dipole trap

laser present. According to theoretical calculations there is a possibility of photoassociation from Sr^+(5s)+Rb(5s) state to Sr(^{1}P)+Rb^+ around $1036\,\mathrm{nm}$[3]. We have not observed such effect.

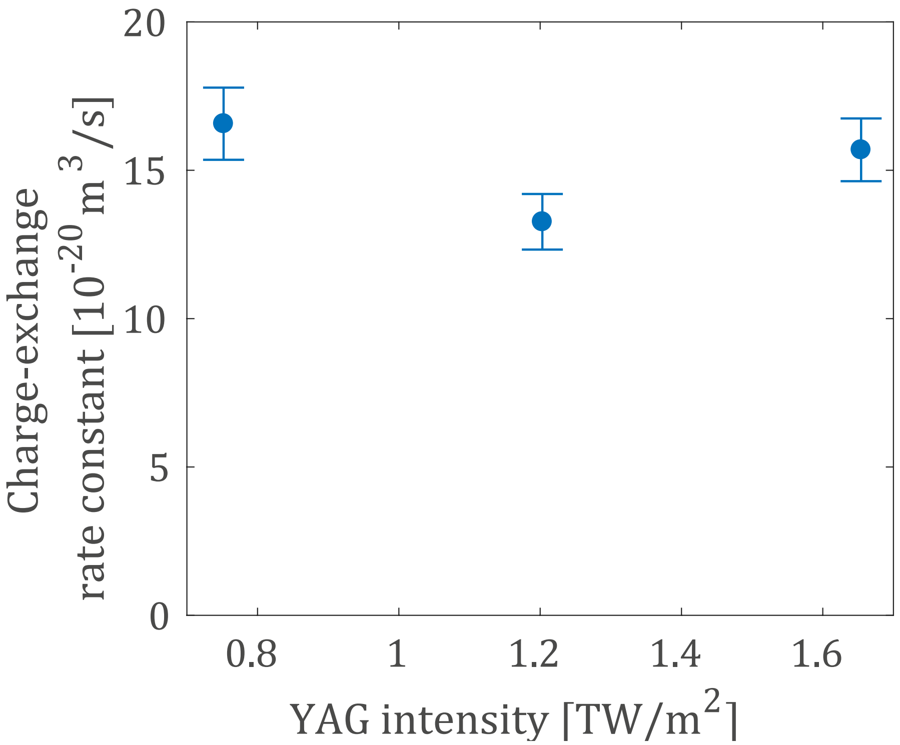

Supplementary Figure 2: **Charge-exchange YAG dependence.** Charge-exchange cross-section at various YAG laser intensities for atoms prepared in a $|1,-1\rangle_{\mathrm{Rb}}$ state. Error bars represent one standard deviation.

Supplementary References

1. Sikorsky, T., Meir, Z., Akerman, N., Ben-shlomi, R. & Ozeri, R. Doppler cooling thermometry of a multilevel ion in the presence of micromotion. *Phys. Rev. A* **96**, 012519 (2017).

2. Meir, Z. *et al.* Dynamics of a ground-state cooled ion colliding with ultracold atoms. *Phys. Rev. Lett.* **117**, 243401 (2016).

3. Aymar, M., Guérout, R. & Dulieu, O. Structure of the alkali-metal-atom strontium molecular ions: Towards photoassociation and formation of cold molecular ions. *J. Chem. Phys.* **135**, 064305 (2011).
